# Diversity and the next-generation physician-scientist

**DOI:** 10.1017/cts.2019.379

**Published:** 2019-07-12

**Authors:** Anit Behera, Jessica Tan, Hanna Erickson

**Affiliations:** 1 MD/PhD Program, Saint Louis University School of Medicine, St. Louis, Missouri, USA; 2 MD/PhD Program, Icahn School of Medicine at Mount Sinai, New York City, New York, USA; 3 Medical Scholars Program, University of Illinois College of Medicine at Urbana, Urbana, Illinois, USA

**Keywords:** Physician-scientist, Diversity

## Introduction

The fields in which physician-scientists work have much to gain by including people with different backgrounds and unique experiences in the search for new knowledge and solutions for existing problems [1,2]. The next generation of physician-scientists will be from the millennial and Gen Z generations, which are far more diverse than previous generations and have the potential to diversify the workforce [3]. Yet, many systemic and cultural barriers exist to limit the entry and advancement of physician-scientists from underrepresented backgrounds [4]. Thus, while addressing the shrinking physician-scientist workforce has been a major focus of the last four decades [5], we argue that promoting diversity in the workforce and reducing barriers for underrepresented groups should also be a priority. Here, we highlight many underrepresented groups that deserve attention and provide suggestions for how to support their inclusion in the physician-scientist workforce.

## Bringing Different Perspectives to the Physician-Scientist Workforce

Historically, a number of groups have been underrepresented in the physician-scientist workforce, including those who identify as female, non-white, or LGBTQ+ and those from a low socioeconomic status (SES) background. Of these groups, women have made the most progress toward equal representation, but even they still have far to go. Since the early 1980s, the proportion of medical graduates that are women has nearly doubled to 48.4% and their representation in the MD-only NIH-funded physician-scientist workforce has increased from 17% in the mid-1990s to 29% in 2012 [4]. A greater disparity for women remains for those with MD and PhD degrees where women made up 42% of MD/PhD program graduates in 2011 and 22% of NIH grant holders with both degrees in 2012 [4]. This disparity is exacerbated for women in leadership positions [6]. Efforts must continue to increase female representation and retention in the physician-scientist pipeline.

Similarly, many non-white groups have been disproportionately underrepresented in the physician-scientist workforce. Together, Hispanic, black, and Native American-identifying persons make up about 30% of the US population [7] but comprised only about 10% of MD/PhD program matriculants in 2018-2019 [8] and just over 5% of NIH-funded physician-scientist investigators [4]. More focus should be placed toward increasing representation of physician-scientists from these groups.

Data are lacking for many other groups including LGBTQ+ and those from a low SES background that should also be represented in the physician-scientist workforce. American culture has come a long way toward allowing people to more comfortably be open about their sexual orientation and gender identity in medicine [9], but many in the LGBTQ+ community still feel marginalized and alienated from medical professionals. Additionally, low SES is a barrier to education at all levels, but there is a particular gap in medical schools. A 2008 analysis by AAMC estimated that 75% of entering medical students have parents in the upper 40% of household income [10]. Additionally, those with lower SES are more likely to have lower MCAT scores [11]. Because MD/PhD program matriculants typically have higher MCAT scores [8], it is likely that this disparity is even more prominent in those pursuing this pathway.

## Broadening the Impact of Physician-Scientists

Traditionally, physician-scientists have selected careers in academia where they chose more cognitive medical specialties and focused their research on the biological mechanisms of diseases [12]. However, physician-scientists are needed both inside and out of academia, from basic to clinical research, both biological and non-biological, and in all medical specialties. It is essential that the next generation of physician-scientists is trained to take on these diverse roles.

Medical doctors both with and without a PhD contribute to the physician-scientist workforce. The latter, while the larger of the two groups, has faced a larger decline [13]. This is also the group with more focus on clinical research than basic or translational research [14]. While this decline could be attributed to a shift toward a more team-based research structure where the number of physician-scientists doing clinical research may be more difficult to track [15], it nonetheless highlights the need for more programs to support the training of physician-scientists in clinical research, particularly in specialties such as surgery that have traditionally been less “research-oriented.”

A notable subgroup of physician-scientists is those who do research in non-traditional areas such as history, English, sociology, anthropology, and public health. These physician-scientists in the social sciences and humanities often dedicate a significant portion of their time to research but are often excluded from discussions of the physician-scientist workforce [4,12]. While they do not fit the traditional definition of a physician-scientist of having a research focus in biological, chemical, or physical science, physician-scientists in the social sciences and humanities are gaining recognition for their ability to contribute to our understanding of many other important facets of medicine [16]. As such, the number of MD/PhD programs supporting training in these areas has dramatically increased in the last decade, but they remain a small portion of all MD/PhD programs [17]. More institutional support for physician-scientist trainees in these areas is needed.

While a majority of physician-scientists follow career paths in academia, they can also make important contributions in other contexts including government/public policy, industry, global health, and science communication. Training programs should be preparing physician-scientist trainees for these other roles during their clinical and research training.

## Recommendations

It is imperative to diversify the physician-scientist workforce to address the needs of a diverse population. There are a number of ways to foster these changes. First and foremost, physician-scientists must be recognized as a diverse population and this diversity must be valued by all stakeholders. The expectations and cultures of training programs, workplaces, professional organizations, and funding agencies need to be more flexible to accommodate and support the retention of physician-scientists and trainees with different needs. This may require changes to tenure and promotion systems or creation or expansion of funding mechanisms that take into account the unique role of the physician-scientist such as the KL2 Mentored Clinical Research Scholar Awards offered through the NIH Clinical and Translational Science Awards Program. Diversification should be considered when deciding to fund training programs as is done for the T32 Medical Scientist Training Program Award offered by the National Institute of General Medical Sciences and also be tracked as an outcome measure of physician-scientist training programs [18]. Institutions can contribute to these efforts by ensuring adequate family medical leave for both trainees as early as medical school and established physician-scientists, as well as addressing gender violence and unconscious bias that can pose significant barriers and can create unsafe work environments. Opportunities for training in non-traditional research areas and settings should be supported by training programs.

Furthermore, these different perspectives need to be better represented, particularly in leadership positions, and be better communicated. A dedicated effort should be made to include qualified underrepresented physician-scientists in speaker line-ups, awardee lists, and other places of honor. Mentors with diverse life experiences are needed to help trainees and early career physician-scientists navigate the unique challenges they may face, including professional and personal ones. Such personal connections can have a strong impact on a mentee’s career trajectory. Formal mentorship programs like the American Physician Scientists Association (APSA) Undergraduate Mentorship Program that pairs undergraduates with current medical students based on demographic, academic, geographic, and other characteristics can facilitate the formation of such mentor–mentee relationships [19].

At the same time, there needs to remain a focus on uniting the physician-scientist community to facilitate sharing of ideas across disparate clinical and research areas. Conferences such as the Association of American Physicians/American Society for Clinical Investigation/APSA Joint Meeting and the Association for Clinical and Translational Science (ACTS) Translational Science Meeting are excellent venues for connecting physician-scientists from different areas. Specific events focused on increasing diversity in the workforce like the Physician Scientist Trainee Diversity Summit being planned by APSA and the Burroughs Wellcome fund can also serve this important function. The interdisciplinary spirit of these meetings needs to be instilled across the whole physician-scientist community.

## Conclusion

As physician-scientist trainees, we are acutely aware of the challenges the next generation of physician-scientists will face but also what they can bring to the workforce. The next generation of physician-scientists has the potential to dramatically diversify the physician-scientist workforce. There is growing interest from underrepresented groups and those with non-traditional career aspirations that must be fostered for the workforce to thrive. More support is needed for trainees and physician-scientists who identify as female, black, Hispanic, and/or Native American. Data are lacking for a number of other underrepresented groups that should also receive more support, including those who identify as LGBTQ+, from low SES backgrounds, involved in non-traditional research areas, and who pursue non-academic careers. It is important that all stakeholders recognize the unique needs and benefits of a diverse physician-scientist workforce and work to reduce barriers to a physician-scientist career. Role models and mentors can have a large impact on an individual’s career, and formal programs like the APSA Undergraduate Mentorship Program may help form these relationships. As the workforce increases in diversity, we must also focus on fostering a community of physician-scientists that values these diverse perspectives.

## References

[1] AlShebli BK , Rahwan T , Woon WL . Ethnic diversity increases scientific impact. arXiv [Internet]. 2018; Retrieved from https://arxiv.org/pdf/1803.02282.pdf.

[2] Gomez LE , Bernet P . Diversity improves performance and outcomes. Journal of the National Medical Association, corrected proof, 2019. doi: 10.1016/j.jnma.2019.01.006 30765101

[3] US Census Bureau. A more diverse nation [Internet]. Retrieved from https://www.census.gov/library/visualizations/2018/comm/diverse-nation.html. Accessed March 17, 2019.

[4] Physician-Scientist Workforce Working Group Report. Bethesda, MD: National Institutes of Health; 2014 Retrieved from https://report.nih.gov/workforce/psw/index.aspx. Accessed June 4, 2019.

[5] Wyngaarden JB . The clinical investigator as an endangered species. The New England Journal of Medicine 1979; 301(23): 1254–1259. 503128 10.1056/NEJM197912063012303

[6] Carr PL , et al. Gender Differences in Academic Medicine: Retention, Rank, and Leadership Comparisons From the National Faculty Survey. Academic Medicine: Journal of the Association of American Medical Colleges 2018; 93(11): 1694–1699. 29384751 10.1097/ACM.0000000000002146 PMC6066448

[7] Humes KR , Jones NA , Ramirez RR . Overview of Race and Hispanic Origin: 2010 [Internet]. U.S. Census Bureau; 2011 (2010 Census Briefs). Report No.: C2010BR-02. Retrieved from https://www.census.gov/prod/cen2010/briefs/c2010br-02.pdf.

[8] FACTS: Applicants, Matriculants, Enrollment, Graduates, MD-PhD, and Residency Applicants Data [Internet]. Association of American Medical Colleges, Data and Analysis. Retrieved from https://www.aamc.org/data/facts/. Accessed February 10, 2019.

[9] Schuster MA . On being gay in medicine. Academic Pediatrics 2012; 12(2): 75–78. 22424395 10.1016/j.acap.2012.01.005

[10] Jolly P. Diversity of U.S. medical students by parental income. AAMC Analysis in Brief 2008; 8(1): 1–2. Retrieved from https://www.aamc.org/download/102338/data/aibvol8no1.pdf. Accessed June 4, 2019.

[11] Grbic D , Jones DJ , Case ST . The Role of Socioeconomic Status in Medical School Admissions: Validation of a Socioeconomic Indicator for Use in Medical School Admissions. Academic Medicine 2015; 90(7): 953. 25629949 10.1097/ACM.0000000000000653

[12] National MD-PhD Program Outcomes Study [Internet]. Washington, DC: AAMC Group on Graduate Research, Education, and Training (GREAT); 2018 Retrieved from https://www.aamc.org/download/489886/data/nationalmd-phdprogramoutcomesstudy.pdf. Accessed February 10, 2019.

[13] Ley TJ , Rosenberg LE . Removing career obstacles for young physician-scientists — loan-repayment programs. The New England Journal of Medicine 2002; 346(5): 368–372. 10.1056/NEJM200201313460515 11821517

[14] Schafer AI , editor. The Vanishing Physician-Scientist? Ithaca: Cornell University Press; 2009.

[15] Bensken WP , et al. Future directions of training physician-scientists: reimagining and remeasuring the workforce. Academic Medicine: Journal of the Association of American Medical Colleges 2019; 94(5): 659–663. 30640263 10.1097/ACM.0000000000002581 PMC6483871

[16] Holmes SM , et al. The first nationwide survey of MD-PhDs in the social sciences and humanities: training patterns and career choices. BMC Medical Education [Internet]. 2017; 17 Retrieved from https://www.ncbi.nlm.nih.gov/pmc/articles/PMC5361808/. Accessed February 11, 2019. 10.1186/s12909-017-0896-1 PMC5361808 28327141

[17] MD/PhD Programs in the Social Sciences & Humanities - American Physician Scientists Association [Internet]. Retrieved from https://physicianscientists.site-ym.com/page/SSH. Accessed February 11, 2019.

[18] Williams CS , et al. Training the physician-scientist: views from program directors and aspiring young investigators. JCI Insight [Internet]. 2018; 3(23). Retrieved from https://insight.jci.org/articles/view/125651. Accessed March 6, 2019. 10.1172/jci.insight.125651 PMC6328016 30518696

[19] Fox BM , Adami AJ , Hull TD . Reinforcing our pipeline: trainee-driven approaches to improving physician-scientist training. Journal of Clinical Investigation 2018; 128(8): 3206–3208. 30010622 10.1172/JCI122100 PMC6063468

